# Analysis of the Effect of Locally Applied Inhomogeneous Static Magnetic Field-Exposure on Mouse Ear Edema – A Double Blind Study

**DOI:** 10.1371/journal.pone.0118089

**Published:** 2015-02-19

**Authors:** Balázs Kiss, János F. László, Andrea Szalai, Róbert Pórszász

**Affiliations:** 1 Department of Biophysics and Radiation Biology and MTA-SE Molecular Biophysics Research Group, Semmelweis University, Budapest, Hungary; 2 Department of Computer Science, University of Debrecen, Debrecen, Hungary; 3 e-Comers LLC, Budapest, Hungary; 4 Department of Pharmacology and Pharmacotherapy, Medical and Health Science Center, University of Debrecen, Debrecen, Hungary; Queen Mary University of London, UNITED KINGDOM

## Abstract

The effect static magnetic field (SMF)-exposure may exert on edema development has been investigated. A 6 h long whole-body (WBSMF) or local (LSMF), continuous, inhomogeneous SMF-exposure was applied on anesthetized mice in an *in vivo* model of mustard oil (MO)-induced ear edema. LSMF was applied below the treated ear, below the lumbar spine, or below the mandible. Ear thickness (*v*) was checked 8 times during the exposure period (at 0, 0.25, 1, 2, 3, 4, 5, and 6 h). The effect size of the applied treatment (*η*) on ear thickness was calculated by the formula *η* = 100% × (1–*v*_j_/*v*_i_), where group *i* is the control group and *j* is the treated group. Results showed that MO treatment in itself induced a significant ear edema with an effect of 9% (*p*<0.001). WBSMF or LSMF on the spine in combination with MO treatment increased ear thickness even further resulting in an effect of *η*>11% in both cases compared to SMF-exposure alone (*p*<0.001). In these cases SMF-exposure alone without MO treatment reduced ear thickness significantly (*p*<0.05), but within estimated experimental error. In cases of LSMF-exposure on the head, a significant SMF-exposure induced ear thickness reduction was found (*η* = 5%, *p*<0.05). LSMF-exposure on the spine affected ear thickness with and without MO treatment almost identically, which provides evidence that the place of local SMF action may be in the lower spinal region.

## Introduction

Only a few studies have been devoted to collect evidence that local exposure to static magnetic field (SMF) may have an effect on edema. Morris and Skalak examined how local, chronic (7 day continuous) exposure to about 120 mT SMF influenced the microvascular response in a murine model [1]. The exposure significantly abrogated the luminal diameter expansion observed in sham-exposed networks. Exposed venular diameter was significantly reduced on day 4 and 7; the arteriolar diameters were significantly reduced on day 7. Venular functional length density was significantly reduced. In another study the authors focused on the direct effect of SMF-exposure under inflammatory conditions [2]. Localized inflammation was induced by the injection of an inflammatory agent λ-carrageenan or histamine into rat hindpaws alone or in conjunction with pharmacological agents. Application of SMF with magnetic induction up to 400 mT for 15 or 30 min immediately following histamine challenge resulted in a significant, 20–50% reduction in edema formation. A 2 h, 70 mT SMF-exposure to λ-carrageenan-induced edema also resulted in a significant (33–37%) edema reduction.

In the present study we also applied an *in vivo* model containing mustard oil (MO)-induced edema of the ear on thiopental anesthetized and thus immobilized mice. We monitored ear thickness 8 times during a 6 h time period post-challenge and set ourselves the following hypotheses: (i) continuous inhomogeneous SMF-exposure affects ear thickness of immobilized, MO-treated mice; consequently, if found true, the effect is exclusively due to magnetic interaction with the living tissues without induced electric phenomena, (ii) SMF application including whole-body exposure or local on the spine, on the head, or on the ear is similarly effective in MO-induced ear edema, (iii) local SMF-exposure on the spine or on the head can replace whole-body exposure in its effect on ear thickness in mice (if any). The magnetic induction values in the exposure conditions of the present work ranged between 2.8 mT and 476.7 mT proven to be optimized for small animal experiments [3,4].

## Materials and Methods

### 1. Static magnetic field-exposure conditions

The whole-body inhomogeneous SMF was generated with an exposure system that was developed, validated, and optimized in multiple animal experiments [3]. The device consisted of 2 ferrous matrices containing 10×10 mm cylindrical neodymium-iron-boron (NdFeB) N50 grade magnets (remanent induction, *B*_r_ = 1.47 T, provided by ChenYang Technologies, Finsing, Germany). The lateral periodicity of the inhomogeneous SMF was 10 mm. The individual magnets in both matrices were placed next to each other with alternating polarity. Magnets facing each other in the 2 matrices were oriented with opposite polarity. The matrices were fixed in a holder where they were separated from each other by a distance of 50 mm realizing an exposure chamber. Anesthetized mice were positioned in this chamber. Magnetic coupling between the matrices provided a magnetic field, in which typical peak-to-peak magnetic induction values along the axis of a magnet in the isocenter were 476.7 mT, 12.0 mT, and 2.8 mT, whereas the average lateral gradient values between 2 neighboring local extremes were 95.3 T/m, 1.2 T/m, and 0.3 T/m at 1.5 mm (target site), 15 mm, and 25 mm from the surfaces of matrices, respectively.

The local inhomogeneous SMF-exposure was accomplished with the system described in e.g., László *et al*. [5] and also similar to the quadrupolar magnet arrangement patented as Method and apparatus for suppressing neuron action potential firings [6]. A single magnetic matrix was used with 2×2 pieces of NdFeB cylindrical magnets (10×10 mm) within. The arrangement of the individual magnets was similar to that in the whole-body exposure system. Measured data along the axis of a magnet were: 476.7 mT peak-to-peak value by 95.3 T/m gradient of the magnetic induction at 1.5 mm (target site), 10 mT by 1 T/m at 10 mm, and 3 mT by 0 T/m at 15 mm. The matrix was positioned below the treated ear of the anesthetized mouse. Thirty two magnetic matrices were submerged into a polystyrene plate upon which mice lied. The upper surface of the magnetic matrices and that of the polystyrene plate were in plane. The matrices were positioned below the treated ear or below the lumbar spine of the anesthetized mouse lying on side, or below the mandible of the anesthetized mouse lying prone.

Sham-exposed animals faced identical exposure conditions, but the matrices included non-magnetized iron cylinders instead of magnets. The time period of continuous SMF-exposure was 6 h. The only intermissions of exposure were when ear thickness measurements took place at certain time points taking less than 30 s each. Magnetic field measurement was performed prior to and separately from the animal experiments with a 5 V calibrated ratiometric linear Hall-effect sensor with 12.3 mV/T sensitivity (model UGN3503, Allegro MicroSystems, MA, USA).

### 2. Edema model, anesthesia

This animal model was described in details by Gábor [7]. One ear of the anesthetized rodent gets smeared with 1% MO (allyl-isothiocyanate) (Sigma-Aldrich, Budapest, Hungary) dissolved in paraffin oil was applied by using a cotton-wool stick locally on one ear of the anesthetized rodent. MO treatment induces a localized inflammation of the ear tissue with a subsequent edema. The action of MO is almost immediate and it peaks at about 3 h post-challenge. The effect spontaneously and gradually ceases in the 6th hour after the challenge.

**Fig 1:**
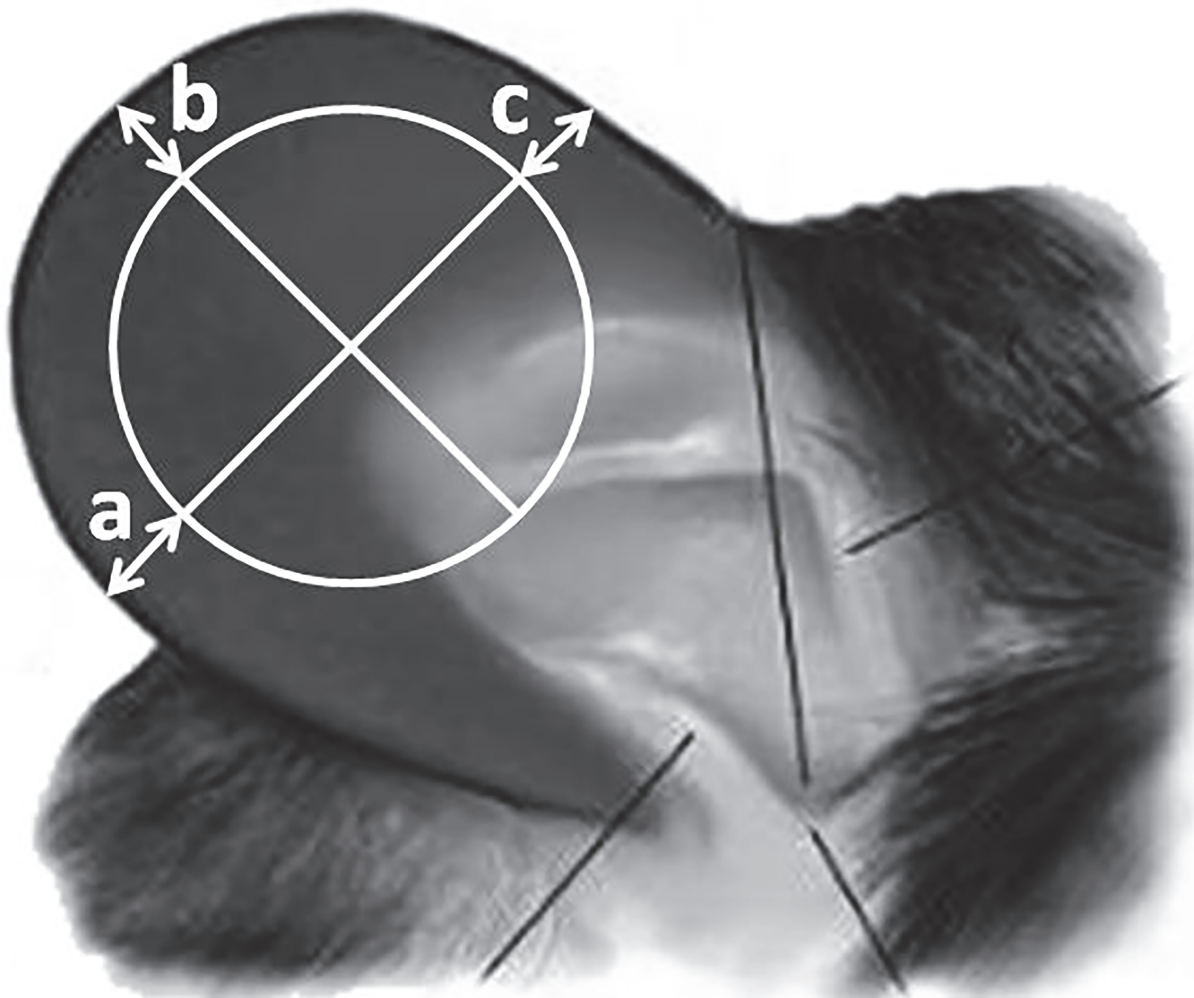
Photo of a mouse *pinna* with the localization of the round cross section of the caliper measuring the ear thickness (photo from [8]).

Edema formation was assessed by the thickness of the treated ear at different time points: at 0 (baseline), at 15 min, at 1 h, and then at every round hour until and including the 6th. A micrometer caliper (Oxford Precision, Leicester, UK) was used to measure ear thickness with a resolution of 0.01 mm. Fig. 1 shows the geometry of the location of the caliper on the pinna of the mouse ear. In the actual measurement, the fitting had to minimize the sum: |*a*–*b*|+|*a*–*c*|+|*b*–*c*|. The residuals of MO were wiped off from the caliper surfaces before measurement. The accuracy of the thickness measurement was estimated to be ±5 μm.

Anesthesia was induced by thiopenthal (Trapanal, Sandoz, Basel, Switzerland) in an amount of 0.2 ml intraperitoneally, repeated in multiples of 0.05 ml as required. The maximum amount given was 0.6 ml; average was 0.29±0.11 ml/mouse (mean±standard error of the mean (SEM)) for the entire experiment.

In order to maintain the core temperature of animals, we used infrared lamps. Temperature on the lab table was set to 37°C and was continuously controlled; rectal temperature of the animals was checked sporadically. During the experiment SMF-exposure did not increase the temperature; SMF is unable to create specific absorption rate.

### 3. Animal groups and treatment options

CD1 (Charles River, Gödöllő, Hungary) male mice (22–44 g, average 33 g) participated in the experiments. Before the experiments they were kept at 22°C, the relative humidity was 30–70%, and the light/dark cycle was 12/12 h. They were maintained on a standard rodent pellet diet and tap water ad libitum. Treatment options were:

1. MO treatment,
2. SMF-exposure including whole-body (WBSMF) treatment, or SMF-exposure on the spine (LSMF spine), on the head (LSMF head), or on the ear (LSMF ear) as compiled in Table 1.

**Table 1:** Relationship between the animal groups and the treatment options.

Group	1	5	9	13	2	6	10	14	3	7	11	15	4	8	12	16
**Mustard oil (MO)**			**x**	**x**			**x**	**x**			**x**	**x**			**x**	**x**
**Static magnetic field (SMF)**		**x**		**x**		**x**		**x**		**x**		**x**		**x**		**x**
**SMF exposure (WB: whole-body; L: local)**	**WBSMF**				**LSMF spine**				**LSMF head**				**LSMF ear**			
**Number of animals**	16	16	15	16	21	23	7	7	16	16	8	8	31	8	8	15
**Numbers of right (treated) ears**	16	15	16	16	0	7	8	7	8	8	8	8	16	8	8	15
**Numbers of left (untreated) ears**	16	0	16	0	28	0	8	0	16	0	16	0	23	0	8	0
**Numbers of ears evaluated**	32	15	32	16	28	7	16	7	24	8	24	8	39	8	16	15
**Application**	negative control to 5 and 9	control to 13	positive control to 13	**edema and SMF reference**	negative control to 6 and 10	control to 14	positive control to 14	**edema and SMF reference**	negative control to 7 and 11	control to 15	positive control to 15	**edema and SMF reference**	negative control to 8 and 12	control to 16	positive control to 16	**edema and SMF reference**

Different control groups were defined as groups either without MO treatment or without SMF-exposure or without both treatment options. Animals were randomly divided into 16 experimental groups. Experiments were carried out at multiple occasions. Untreated left ear thickness served as self-control in some experiments and was evaluated according to its treatment (Table 1). Note that the sum of all animals exceeds half of the sum of ears evaluated, because at some occasions only treated ears were monitored. This is why odd numbers of evaluated ears may occur. Five animals died premature, their partial data were excluded from the evaluation; these animals were considered drop-outs.

### 4. Ethics

Altogether 231 naïve mice participated in the series of experiment; 84 animals got mustard oil treatment, 109 animals were exposed to SMF. Anesthetized mice in each group were sacrificed by rapid decapitation performed with surgical scissors subsequent to the experiments. Animals were treated in accordance with the European Communities Council Directives (86/609/ECC) and the Hungarian Act for the Protection of Animals in Research (XXVIII tv. 32). The experiments were in harmony with the Ethical Codex of Animal Experiments and were approved by the Hungarian Ethical Committee for Animal Research at University of Debrecen (permission number: 2/2007/DEMÁB).

### 5. Blinding

A quasi-randomly numbered list of devices determined which 16 of the 32 devices used simultaneously was magnetized. Care was taken to have a balanced list. No lab personal was aware, whether a device was magnetic or not. No magnetizable or magnetically anisotropic materials were allowed in the lab during the experiments. The devices were built in polystyrene plates in advance with their number in ascending order. The numbered list was disclosed to the statistician only after he had finished the calculations based on group numbers.

### 6. Statistical analysis

The primary outcome measure in the present experimental series was ear thickness *v*(*s,m,a,r,t,d,e,n,b*) defined as a 9 dimensional quantity depending on

- *s* = 0 or 1 side (treated right or untreated left),
- *m* = 0 or 1 MO treatment (no or yes),
- *a* = 0 or 1 magnetization (no or yes, 0 means sham-exposure),
- *r* = 0, 1, 2, or 3 treatment option (whole-body SMF, local SMF on the ear, on the head, or on the spine),
- *t* = 0, 0.25, 1, 2, 3, 4, 5, or 6 h time points of data acquisition during a measurement,
- *d* = 0, 1, 2, 3, 4, 5, 6, or 7 date of experiment (between 20 October, 2010 and 21 March, 2013),
- *e* quantity of intraperitoneally administered anesthetics thiopental depending on *t*,
- *n*[1;231] identification number of animal so that Σ_s_ *n*_s_ = Σ_m_ *n*_m_ = Σ_a_ *n*_a_ = Σ_r_ *n*_r_ = Σ_t_ *n*_t_ = Σ_d_ *n*_d_ = Σ_e_ *n*_e_ = Σ_b_ *n*_b_ = *n*.
- *b* body mass of the animal in g.

The effect size (*η*) between group *i* and *j* was defined as *η*_ij_ = 100% × (1–*v*_j_/*v*_i_). Group *i* is usually the negative or the positive control group, *j* is usually a treated group (SMF-exposure alone, or in combination with MO). According to this definition, effect size could be negative. Negative *η* meant ear thickness aggravation as a possible consequence of edema development.

Due to kind of exposure and the definition of groups, the inherent equal SMF treatment of ears *v*(0,0,*a,r,t,d,e,n,b*) = *v*(1,0,*a,r,t,d,e,n,b*) applied for *r* = 0, 1, and 3.

First the normality of the *v* data series was checked by Kolmogorov-Smirnov test. For baseline comparison single factor ANOVA was used. Two-factor rANOVA was used in case of time dependent data comparison in multiple groups, where one factor was the recording time points of the experiment, the other was the treatment options. If we found significant differences in the multiple group analysis, we applied the Games-Howell post hoc test to identify significant differences between binary group averages of *v*. This test is not sensitive to unbalanced groups. If significance was established, effect sizes in percent (*η*) and the corresponding probability values (*p*) are shown in the text. *p*<0.001 values are not provided numerically.

Altogether 2178 data values have been evaluated (726 WBSMF, 436 LSMF on the spine, 464 LSMF on the head, 552 LSMF on the ear), 182 data points were missing. These latter have been substituted by (i) time averages of the nearest neighbors’ data, if both neighbors existed, (ii) group averages otherwise.

## Results

**Fig 2:**
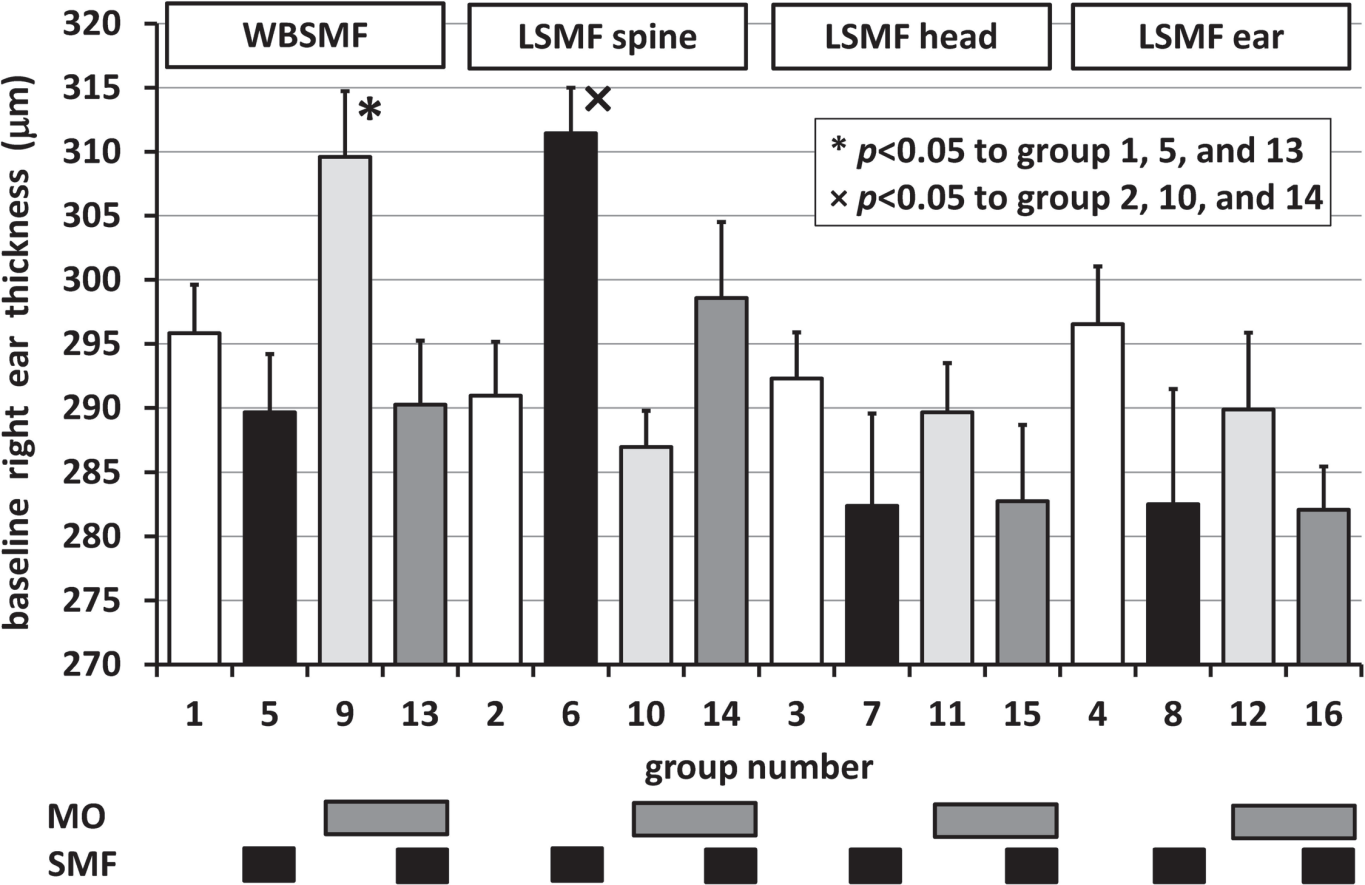
Baseline right ear thickness in μm for different groups (*c.f.*, Table 1). * and × refer to significant differences in multiple groups to Groups 1, 5, 13 and to Groups 2, 10, 14, respectively. Bars above the columns denote treatment types. Error bars show positive standard errors of the mean (SEM).

Significant differences in the baseline right ear thickness values (*v*(0,*m,a,r*,0,*d,e,n,b*)) were revealed for WBSMF and LSMF in the spine groups as shown in Fig. 2. Random distribution of animals into groups resulted in an unfortunately high baseline average right ear thickness in Group 6 compared to Groups 2, 10, and 14, and also in Group 9 compared to Groups 1, 5, and 13. In these 2 groups baseline correction was carried out so that *v*(0,*m,a,r*,0,*d,e,n,b*) = 1/*n*_r_*Σ_i_ *v*(0,*m,a,i*,0,*d,e,n,b*) for all *r*’s (*i* = 0, 1, 2, and 3).

No significant differences occurred between left and right ears within the first 15 min (*p*≥0.152). An inherent presumption in our experiments was that there was no cross talk between the ears (*t*>15 min). This was supported by the fact that the minimum *p* value (in case of local exposure on the ear) in comparing sham-exposed left and right ear thicknesses was 0.085 (–3.0% meaning thicker left ear than right) beyond 15 min experimental time. However, when SMF-exposure was applied whole-body or on the head, significant differences occurred: for WBSMF an effect of –3.8% (*p* = 0.025) and for LSMF on the head 2.9% (*p* = 0.037) was measured. In these cases we did not pool the data of different sides meaning *v*(*s,m,a*,0,*t,d,e,n,b*)≠1/*n*_d_*Σ_j_ *v*(*s,m,a*,0,*t,j,e,n,b*) and *v*(*s,m,a*,2,*t,d,e,n,b*)≠1/*n*_d_*Σ_k_ *v*(*s,m,a*,2,*t,k,e,n,b*) for all *d*’s [*j,k*]∈[0, 1, 2,…, 7].

We also checked, whether a cross talk would appear between left ears of animals, whose right ear was treated exclusively with MO and those that did not get MO treatment. No such case was observed beyond 15 min experimental time, when MO on the right ear would have increased the ear thickness of the left ear.

Significant differences in the baseline right ear thickness values (*v*(*t* = 0)) were revealed for WBSMF and LSMF on the spine groups as shown in Fig. 2. Random distribution of animals into groups resulted in an unfortunately high baseline average ear thickness in Group 5 compared to Groups 1, 9, and 13, and also in Group 10 compared to Groups 2, 6, and 14. In these 2 groups baseline correction was carried out. No significant differences occurred between left and right ears within the first 15 min (*p*_min_ = 0.152).

We estimated the systematic error of the measurement by evaluating the ear thickness evolution throughout the 6 h of the experiments under sham-exposure condition. A small increase of 3.60 μm was observed that would not require a correction. Another potential hazard for the accuracy of the measurement was the applied anesthesia. We checked, whether thiopental injection modified ear thickness in itself. In case of 20 animals treated as negative control (4 from WBSMF, 8 from LSMF ear, 8 from LSMF head group) an exact monitoring of thiopental administration (function *e*(*t*)) was carried out. We normalized *e*(*t*) to body mass. Then, we correlated the normalized *e*(*t*) with the average ear thickness of these animals (1/*n*_e_*Σ_l_ *v*(*s,m,a,r,t,d,l,n,b*)). The Pearson correlation coefficient was 83.3%. As a result of these observations we could define a maximum effect of ±1.2% as the systematic error in the measurement of ear thickness. The caliper reading error was ±2% (average ear thickness of 305 μm). Altogether an estimated maximum experimental error was ±3.1%.

**Fig 3:**
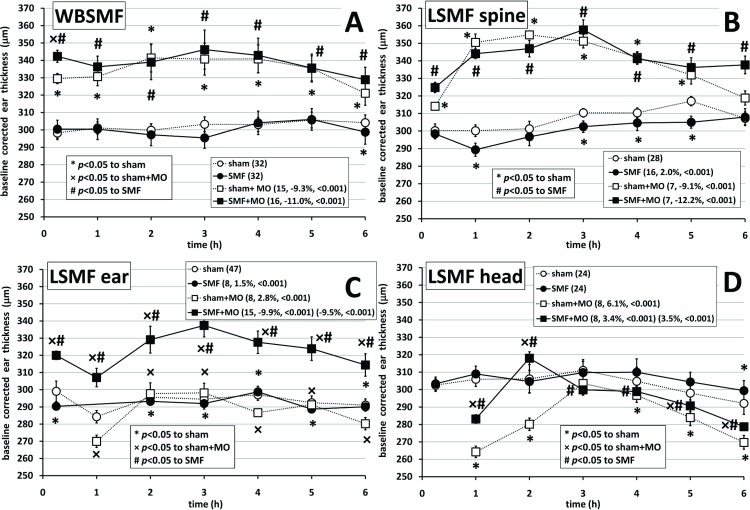
Evolution of average ear thickness in μm as a function of experimental time (h) for A) whole-body static magnetic field (WBSMF)-exposure, B) local (LSMF)-exposure on the spine, C) LSMF-exposure on the ear, and D) LSMF-exposure on the head. Treatment options appear in the legend: for sham: the number in the parenthesis is the ear number evaluated in the group (*n*). For SMF only and for sham+mustard oil (MO): the first number in the parenthesis is *n*, the second number (if exists) is the effect *η*, the third number (if exists) is the probability of significant difference (*p*) compared to negative control for the complete 6 h time period. For SMF+MO: the first number in the first parenthesis is *n*, the second number is *η*, the third number is *p* compared to SMF alone; the first number in the second parenthesis (if the second parenthesis exists) is *η*, the second number is *p* compared to positive control. *, ×, and/or # above or below the markers show significant differences to negative, positive controls, and/or SMF alone, respectively at the specific time point. Lines connecting markers guide the eye only. Error bars show standard errors of the mean (SEM). All differences were estimated by Games-Howell *post hoc* tests.

When evaluating ear thickness (baseline corrected where appropriate) on multiple groups, two-way rANOVA always resulted in *p*<0.001 for time evolutions, for different treatments, and for interactions as well. Ear thickness as a function of experimental time (measured at 0.25, 1, 2, 3, 4, 5, and 6 h following MO challenge) is shown in Fig. 3A, B, C, and D. The figure title refers to the method (and place) of SMF application (panel A: whole-body; panel B: local on the spine; panel C: local on the right ear; panel D: local on the head). Number of ears evaluated in the group, significant effects compared to negative control, to positive control, and to SMF-exposure alone, are shown in the figure. Negative effect means the enhancement of ear thickness, in case of MO treatment most likely due to edema formation.

In cases of LSMF head and ear treatments we failed to reproduce the ear edema model according to the protocol 6; all the more so, MO treatment in these arrangements seemed to even induce significant ear thickness attenuation rather than an edema in the first 2 h of the experiment. Since we must attribute this to an experimental artifact, we do not discuss these situations beyond the effect of SMF-exposure on sham-exposure.

**Fig 4:**
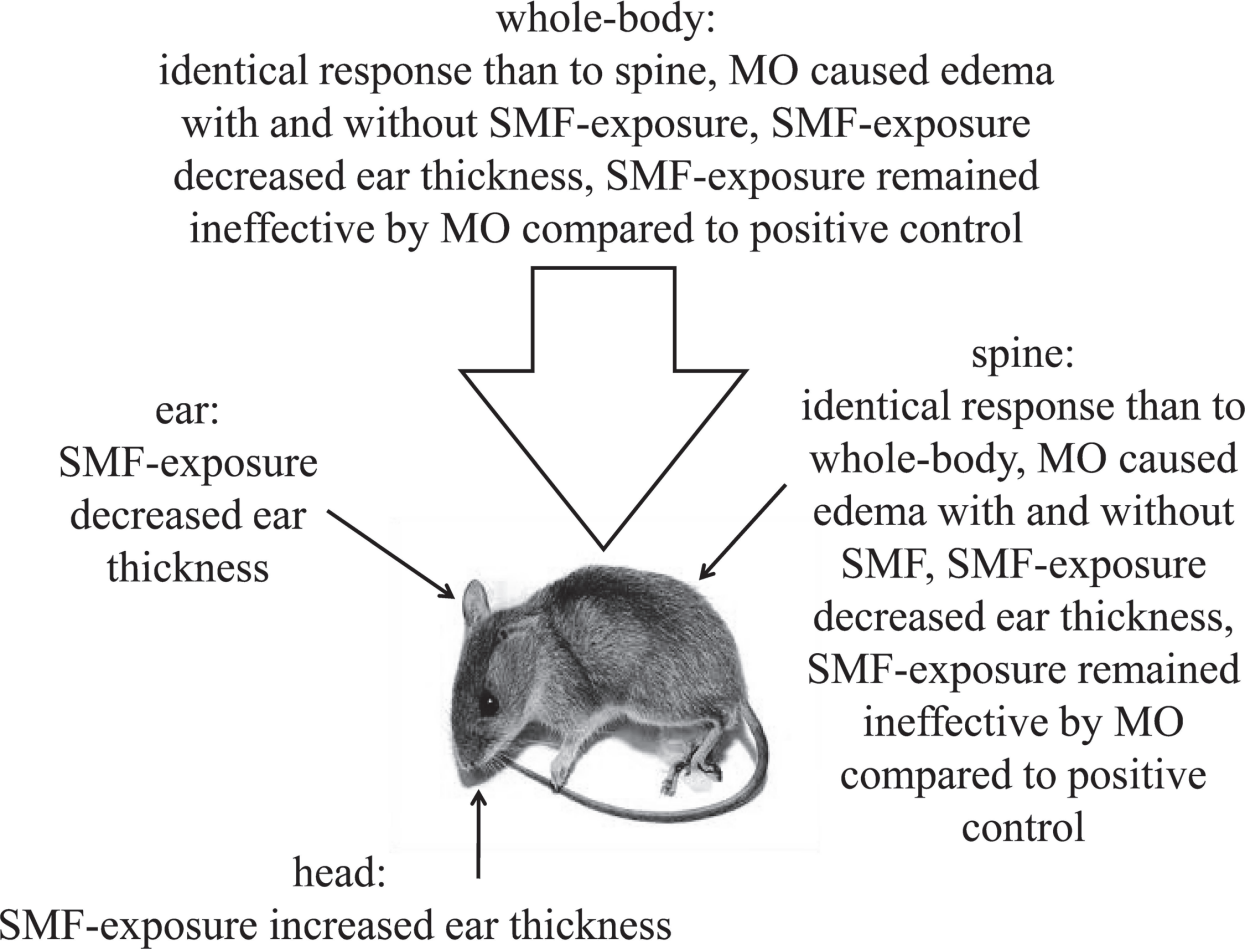
The observations in mice challenged with mustard oil (MO) in an induced ear edema model by different static magnetic field (SMF)-exposures: whole-body, local on the spine, local on the ear, and local on the head.

In none of the 4 exposure situations (WBSMF, LSMF on the spine, ear, and head) could we detect a clinically significant effect (statistically significant and exceeds experimental error) of SMF-exposure on the ear thickness of mice, see Fig. 3A-D. The highest effect was 2% by p<0.001 in case of LSMF applied on the spine. WBSMF (Fig. 3A) and LSMF on the spine (Fig. 3B) treatment options had almost identical effects on the mice ear. In these cases MO treatment in itself caused a significant raise in the ear thickness by *η*>9% (*p*<0.001) in harmony with the literature [7]. MO treatment combined with SMF-exposure affected ear thickness even stronger, increased it by *η*>11% (*p*<0.001) compared to SMF-exposure alone. In combination with MO treatment there was hardly any observable trend attributable to SMF-exposure compared to positive control in case of LSMF on the spine only. The observations are compiled in Fig. 4.

## Discussion

MO could have activated non-selective cation channels in the central nervous system so that the action of SMF-exposure would have aggravated. This pathway could be one of the TRP channels, since TRPs are widely distributed in the brain [9]. Most likely this was not the case in the present experiments in the brain, but further investigation is needed in order to verify this scenario.

Thiopental is being known to induce or aggravate edema in already pre-stressed animals: co-administration of pentoxiphylline and thiopental may even cause acute lethal lung edema in rats [10]. Still, we could not reveal any vasodilational effect that could be associated with thiopental in our present experiments. No significant effect of the anesthetics was found on the thickness of the ear of the mouse.

More importantly, the target site was 1.5 mm from the magnetic surface and the measured magnetic inductions at the target site were identical for all sites (ear, head, and spine). In an earlier study, intracerebroventricularly administered naloxone did not provide the same antagonizing effect against the SMF-induced inhibition of pain as the subcutaneously administered naloxone [11]. The authors’ conclusion then was that the analgesic effect found may have likely not been at the supraspinal level. The fact that whole-body SMF-exposure and local SMF-exposure on the spine resulted in practically identical ear thicknesses and significant effects of the SMF may involve a lower spinal response to the SMF-exposure as suggested earlier by Gyires *et al*. [11].

The observed decrease of ear thickness under SMF-exposure compared to sham-exposure must have been an effect of hypotension [12] or vasoconstriction [13] described earlier.

MO treatment was verified to induce an ear edema at least in case of WBSMF and LSMF on the spine. MO, like several other environmental irritants targets transient receptor potential (TRP) 1A ion channels on primary afferent nociceptors thus causing Ca^2+^ influx and phospholipase C activation [14]. A study showed that 150 s exposure to 125 mT homogeneous SMF significantly altered the kinetics of voltage gated Na^+^ channels [15]. A time-varying magnetic field was shown to induce intracellular Ca^2+^ release in cultured cells [16]. Thirty minutes exposure to an inhomogeneous SMF significantly diminished the number of both intraperitoneally administered acetic acid- and Epsom salt-induced abdominal contractions (acute visceral nociception), formalin-evoked paw lickings and liftings in both phase I (acute somatic nociception) and phase II (acute inflammatory nociception), and mechanical hyperalgesia evoked by intraplantar injected TRPV1 capsaicin receptor agonist resiniferatoxin [16]. Significant inhibitory effects of SMF-exposure on formalin-induced nociception and carrageenan-evoked hyperalgesia were absent in resiniferatoxin-pretreated mice [16]. Based on these observations it is conceivable that MO and SMF act on similar targets in nociceptors and may produce a non-linear interaction that can be beneficially or aggravatingly superposed. Microvascular tone was also found to be affected by the SMF with field strength of several mT as demonstrated by several workgroups [17,18]. Locally applied SMF in rabbits caused increased vasoconstriction when the vascular tone was low in their microcirculation but under noradrenaline-induced high vascular tone SMF was found to enhance vasodilation with increased vasomotion [18]. Localized SMF demonstrated a biphasic, modulatory effect on microvascular tone in rats by causing vasoconstriction in previously vasodilated arterioles and resulting in vasodilation of vessel with increased tone [19]. When prolonged compressive mechanical loading was applied to the cutaneous tissue of rats under anesthesia significant increase was found in the endothelial related metabolic activity in the stressed skin whereas SMF did not induce significant changes in the unstressed skin [20]. These observations imply the possibility that SMF in our present study could aggravate the MO-induced edema by enhancing the vasodilation.

The SMF in the present experiments was strongly inhomogeneous. In principle, this allowed for induced magnetic potential differences in points of the body of the animal 10 mm apart according to the lattice constant of the closed packed magnets. This magnetic pattern covered an area of 40×40 mm on the ear, on the head, or on the lumbar spine. It was suggested earlier for both homogeneous [3,21] and inhomogeneous SMF [3,22] that the self-motion of rodents through an SMF may have increased the chances of occurrence of induced electric effects that conclusively, may have distorted the quantitative observations of biological responses thought to be exclusively associated with the SMF-exposure. If animals are paralyzed during the experiment though, self-motion has a negligible contribution to biological responses. However, motion of ion currents within the body of anesthetized mice may still induce flow potentials. These potentials are supposed to have a measurable effect on responses only in the range of magnetic induction above 8 T [23].

SMF-exposure on the head basically failed to perform an observable effect on ear thickness of the mouse. However, if challenged by MO, the ear thickness responded in a significantly aggravating manner compared to the unprovoked ear. This suggests that central exposure indeed acts *via* channels (TRP, opiates [11], and/or cytokines [24]) implied in inflammatory reactions and fails to affect the biological response otherwise. More surprisingly, exposure at the lumbar spine provided contrary effects: an expressed aggravation on the unchallenged ear thickness and hardly any effect on the MO treated ear. It seems that spinal exposure invokes a response alone lacking other challenges, which provides evidence that SMF-exposure acts in the lumbar spinal region first of all and can excite materials or signals even under physiological circumstances.

It has been demonstrated that cognitive processes involving visual distraction [25] or high working memory load [26] may modulate thermal pain perception. High-resolution functional magnetic resonance imaging revealed decreased neuronal response to painful thermal stimulations by the inhibition of incoming pain signals in the dorsal horn of the cervical spinal cord when painful stimuli were applied simultaneously with the memory load [26]. One main aim in our present study was to minimize the role of the cognitive processes during pain perception, therefore we applied local anesthesia on mice. Neuronal response on mustard oil-induced colon inflammation of rats anesthetized with pentobarbital involves specific post-synaptic dorsal column cells [27], the activation of these cells occurs regardless to the applied anesthesia. Based on these observations it is plausible that the static magnetic field in our experiments applied locally to the brain acts similar to the memory load most likely *via* non-specific brain activation by ion channel modulation. Extremely low-frequency electromagnetic fields (ELF-EMF) have been shown to modulate Ca^2+^ and Na^+^ channels in rat cerebellar granule cells in a similar way as the intracellular application of arachidonic acid and prostaglandin E2 [28].

In the study of Morris and Skalak the single magnet was fixed 2 mm above the 5 mm thick hindpaw of the anesthetized rat [1]. Magnets provided 7.5, 50, or 250 mT at the target site, single exposure times (immediately following the challenge) were 15, 30, or 120 min. The edema monitoring went on for 210 min post-challenge. SMF-exposure of 50 mT for 15 or 30 min did not affect λ-carrageenan-induced, but significantly reduced histamine-induced edema formation. A 2 h long exposure with 50 mT SMF was enough to decrease a reduced dose of λ-carrageenan-induced edema. Fifteen minutes exposure with 250 mT SMF failed to influence histamine-induced edema. Since the co-administration of L-arginine (a nitric oxide agonist) with histamine followed by a 15 min 50 mT SMF-exposure resulted in a significant reduction in the L-arginine-potentiated edema, but not to the level of SMF treated histamine alone, and the concurrent administration of BAY K 8644 (a calcium channel agonist) with histamine followed by a 50 mT SMF-exposure resulted in the abolition of the initial edema reduction observed with SMF-exposure, the authors suggested that SMF application may act via L-type Ca^2+^ channels and not via nitric oxide signaling in histamine-stimulated paws [1]. All these observations suggest that SMF-exposure should act against the formation or the evolution of an edema. Interestingly, Morris and Skalak [2] found that the application of a 400 mT SMF had no effect on histamine-induced edema. Accordingly, they suggested an upper limit of magnetic induction for edema suppression.

Both carrageenan and MO are transient receptor potential ankyrin 1 (TRPA1) agonists. However, histamine acts on its own receptors H1-H5. Mast cells contain TRPA1 (and transient receptor potential vanilloid 1 (TRPV1)) receptors; consequently, MO is capable of depleting histamine. Histamine on the other hand, does not play a rule in the direct stimulation of TRPA1 receptors. As we know from the recent review of Alves *et al*. [29], extracellular adenosine triphosphate secreted from mast cells and granulocytes can activate certain P2X receptors, ion channels. These ion channels may then provoke vasodilatation through nitric oxide (NO) signal paths. SMF-exposure seems to be able to block this path. Carrageenan also acts along this path. Purinergic receptor P2X7R agonists as well as MO activate nociceptive neurons in the dorsal horn using different signals paths [29]. We can hypothesize that supposed SMF-exposure acts similarly, a positive feed-back mechanism may generate an antinociceptive effect.

In response to hypotheses (i) and (ii): continuous inhomogeneous, whole-body or local SMF-exposure on the spine affected ear thickness in mice immobilized by anesthesia compared to negative or positive control, but the effects remained below experimental error. The measured effects were exclusively due to magnetic interaction with the living tissues without induced electric phenomena. In response the hypothesis (iii): local SMF-exposure on the spine can replace whole-body exposure in its effect on ear thickness in mice within the ear edema model. It seems that within this model the place of action of the SMF-exposure is in the lower spinal region.

## Supporting Information

S1 Table The table provides raw, untransformed data of ear thickness values, biometric parameters and exposure conditions for individual animals. (XLSX)

## References

[1] Morris CE, Skalak TC. Chronic static magnetic field exposure alters microvessel enlargement resulting from surgical intervention. J Appl Physiol. 2007;103 (2): 629–36. 17478604 10.1152/japplphysiol.01133.2006

[2] Morris CE, Skalak TC. Acute exposure to a moderate strength static magnetic field reduces edema formation in rats. Am J Physiol Heart Circ Physiol. 2008;294 (1): H50–7. 17982018 10.1152/ajpheart.00529.2007

[3] László J, Reiczigel J, Székely L, Gasparics A, Bogár I, et al Optimization of static magnetic field parameters improves analgesic effect in mice. Bioelectromagnetics. 2007;28 (8): 615–27. 17654477 10.1002/bem.20341

[4] Kiss B, Gyires K, Kellermayer M, Laszlo JF. Lateral gradients significantly enhance static magnetic field-induced inhibition of pain responses in mice—a double blind experimental study. Bioelectromagnetics. 2013;34: 385–396. 10.1002/bem.21781 23737187

[5] László JF, Farkas P, Reiczigel J, Vágó P. Effect of local exposure to inhomogeneous static magnetic field on stomatological pain sensation—A double-blind, randomized, placebo-controlled study. Int J Radiat Biol. 2012;88 (5): 430–8. 10.3109/09553002.2012.661916 22288770

[6] Holcomb RR. Method and apparatus for suppressing neuron action potential firings. USA Patents (US 5312321 A). 1994.

[7] Gábor M. Models of acute inflammation in the ear In: Winyard PG, Willoughby DA, editors. Inflammation protocols. New York: Humana Press; 2003 pp. 129–37.

[8] Waltham MA. Comparative Anatomy and Histology: A Mouse and Human Atlas Amsterdam: Elsevier; 2012.

[9] Moran MM, Xu H, Clapham DE. TRP ion channels in the nervous system. Curr Opin Neurobiol. 2004;14 (3): 362–9. 15194117 10.1016/j.conb.2004.05.003

[10] Pereda J, Gomez-Cambronero L, Alberola A, Fabregat G, Cerda M, et al Co-administration of pentoxifylline and thiopental causes death by acute pulmonary oedema in rats. Br J Pharmacol. 2006;149 (4): 450–5. 16953192 10.1038/sj.bjp.0706871 PMC1978439

[11] Gyires K, Zádori ZS, Rácz B, László J. Pharmacological analysis of inhomogeneous static magnetic field-induced antinociceptive action in the mouse. Bioelectromagnetics. 2008;29 (6): 456–62. 10.1002/bem.20413 18435453

[12] Okano H, Ohkubo C. Exposure to a moderate intensity static magnetic field enhances the hypotensive effect of a calcium channel blocker in spontaneously hypertensive rats. Bioelectromagnetics. 2005;26 (8): 611–23. 16189831 10.1002/bem.20144

[13] Okano H, Ohkubo C. Modulatory effects of static magnetic fields on blood pressure in rabbits. Bioelectromagnetics. 2001;22 (6): 408–18. 11536282 10.1002/bem.68

[14] Bautista DM, Jordt SE, Nikai T, Tsuruda PR, Read AJ, et al TRPA1 mediates the inflammatory actions of environmental irritants and proalgesic agents. Cell. 2006;124 (6): 1269–82. 16564016 10.1016/j.cell.2006.02.023

[15] Rosen AD. Effect of a 125 mT static magnetic field on the kinetics of voltage activated Na^+^ channels in GH3 cells. Bioelectromagnetics. 2003;24 (7): 517–23. 12955757 10.1002/bem.10124

[16] Sándor K, Helyes Z, Gyires K, Szolcsányi J, László J. Static magnetic field-induced anti-nociceptive effect and the involvement of capsaicin-sensitive sensory nerves in this mechanism. Life Sci. 2007;81 (2): 97–102. 17568617 10.1016/j.lfs.2007.04.029

[17] Ohkubo C, Xu S. Acute effects of static magnetic fields on cutaneous microcirculation in rabbits. In Vivo. 1997;11: 221–225. 9239515

[18] Okano H, Gmitrov J, Ohkubo C. Biphasic effects of static magnetic fields on cutaneous microcirculation in rabbits. Bioelectromagnetics. 1999;20: 161–171. 10194558 10.1002/(sici)1521-186x(1999)20:3<161::aid-bem2>3.0.co;2-o

[19] Morris C, Skalak T. Static magnetic fields alter arteriolar tone in vivo. Bioelectromagnetics. 2005;26: 1–9. 15605401 10.1002/bem.20047

[20] Li Z, Tam EW, Mak AF, Lau RY. Wavelet analysis of the effects of static magnetic field on skin blood flowmotion: investigation using an in vivo rat model. In Vivo. 2007;21: 61–68. 17354615

[21] László J, Gyires K. 3 T homogeneous static magnetic field of a clinical MR significantly inhibits pain in mice. Life Sci. 2009;84 (1–2): 12–7. 19000698 10.1016/j.lfs.2008.10.009

[22] (a) László J. External static magnetic field as a device for self-motion perception—A pathophysiological rodent model and its consequences In: Costa EV, editor. Horizons in Neuroscience Research. Volume 5 Hauppauge: Nova Science Publishers; 2011 pp. 106–24.

[23] Jokela K, Saunders RD. Physiologic and dosimetric considerations for limiting electric fields induced in the body by movement in a static magnetic field. Health Phys. 2011;100 (6): 641–53. 10.1097/HP.0b013e318202ec7e 22004933

[24] Vergallo C, Dini L, Szamosvolgyi Z, Tenuzzo BA, Carata E, et al In vitro analysis of the anti-inflammatory effect of inhomogeneous static magnetic field-exposure on human macrophages and lymphocytes. PLoS One. 2013;8 (8): e72374 10.1371/journal.pone.0072374 23991101 PMC3753352

[25] Valet M, Sprenger T, Boecker H, Willoch F, Rummeny E, et al Distraction modulates connectivity of the cingulo-frontal cortex and the midbrain during pain—an fMRI analysis. Pain. 2004;109 (3): 399–408. 15157701 10.1016/j.pain.2004.02.033

[26] Sprenger C, Eippert F, Finsterbusch J, Bingel U, Rose M, et al Attention modulates spinal cord responses to pain. Curr Biol. 2012; 22 (11): 1019–22. 10.1016/j.cub.2012.04.006 22608507

[27] Al-Chaer ED, Westlund KN, Willis WD. Sensitization of postsynaptic dorsal column neuronal responses by colon inflammation. Neuroreport. 1997;8 (15): 3267–73. 9351655 10.1097/00001756-199710200-00016

[28] He YL, Liu DD, Fang YJ, Zhan XQ, Yao JJ, et al Exposure to extremely low-frequency electromagnetic fields modulates Na^+^ currents in rat cerebellar granule cells through increase of AA/PGE2 and EP receptor-mediated cAMP/PKA pathway. PLoS One. 2013;8 (1): e54376 10.1371/journal.pone.0054376 23349866 PMC3551899

[29] Alves L, Bezerra R, Faria R, Ferreira L, da Silva Frutuoso V. Physiological roles and potential therapeutic applications of the P2X7 receptor in inflammation and pain. Molecules. 2013;18: 10953–72. 10.3390/molecules180910953 24013409 PMC6270334

